# *iBehavior*—a preliminary proof of concept study of a smartphone-based tool for the assessment of behavior change in neurodevelopmental disabilities

**DOI:** 10.3389/fpsyg.2023.1217821

**Published:** 2023-10-18

**Authors:** Andrew Dakopolos, Dana Glassman, Haleigh Scott, Michael Bass, David Hessl

**Affiliations:** ^1^MIND Institute, University of California Davis Health, Sacramento, CA, United States; ^2^Department of Psychiatry and Behavioral Sciences, University of California Davis School of Medicine, Sacramento, CA, United States; ^3^Department of Psychiatry and Behavioral Sciences, UC Davis MIND Institute, Sacramento, CA, United States; ^4^Fienberg School of Medicine, Northwestern University, Chicago, IL, United States

**Keywords:** developmental disabilities, ecological momentary assessment, EMA, behavior tracking, Down syndrome, fragile X syndrome

## Abstract

**Purpose:**

The purpose of the present study was to describe the content and function of *iBehavior*, a smartphone-based caregiver-report electronic ecological momentary assessment (eEMA) tool developed to assess and track behavior change in people with intellectual and developmental disabilities (IDDs), and to examine its preliminary validity.

**Methods:**

Ten parents of children (ages of 5–17 years) with IDDs (*n* = 7 with fragile X syndrome; *n* = 3 with Down syndrome) rated their child’s behavior (aggression and irritability, avoidant and fearful behavior, restricted and repetitive behavior and interests, and social initiation) using *iBehavior* once daily for 14 days. At the conclusion of the 14-day observation period, parents completed traditional rating scales as validation measures, as well as a user feedback survey.

**Results:**

Across the 140 possible observations, 8 were skipped, leading to a 94% response rate over 10 participants’ observation periods. Participants also completed 100% of items for each of their logged observations. Parent ratings using *iBehavior* showed emerging evidence of convergent validity among domains with traditional rating scales including the Behavior Rating Inventory of Executive Function 2 (BRIEF-2), and Aberrant Behavior Checklist-Community (ABC-C). *iBehavior* was feasible in the sample, and parent feedback indicated high overall satisfaction.

**Conclusion:**

Results of the present pilot study indicate successful implementation and preliminary feasibility and validity of an eEMA tool for use as a behavioral outcome measure in IDDs.

## 1. Introduction

The measurement of behavior, internal states, cognition, and experiences of people with intellectual and developmental disabilities (IDDs) is complicated by many methodological challenges. Although approaches for self-report among individuals with IDDs are emerging in order to minimize almost exclusive reliance on proxy-reporting (Fisher et al., 2014; Kaiser and Halvorsen, 2022; Kenworthy et al., 2022; Santoro et al., 2022), barriers related to communication, insight, and cognitive functioning persist. Thus, traditional retrospective proxy-report questionnaires remain the prominent approach to characterize behavior (e.g., Aman et al., 1985; Esbensen et al., 2003; Sparrow et al., 2016), as diagnostic supplements (e.g., Rutter et al., 2003; Conners, 2008; Constantino and Gruber, 2012), and in clinical trials as primary outcomes (Esbensen et al., 2017; Thurm et al., 2020) for those with IDDs.

When applied rigorously, proxy approaches are psychometrically supported, well-validated, and cost-effective (Brown et al., 2002). Despite these benefits, proxy-reported questionnaires can produce rater estimates of behavior prone to systematic biases (Shiffman et al., 2008). Retrospective reporting has been shown to be susceptible to recall bias (Zhang et al., 2017), and can be influenced by factors such as the salience or outcome of the behavior (Erickson and Jemison, 1991), as well as the rater’s mental or emotional state at the time of rating (Clark and Teasdale, 1985; Strongman and Russell, 1986). Raters may also over- or under-estimate their ratings, particularly in those with IDDs (Chandler et al., 2016). In particular, parents of children with IDD have been shown to overestimate their child’s overall functioning (Chandler et al., 2016). Furthermore, while many proxy-reported measures typically include some standardized instructions, there is usually no rater training and little communication between experimenter and rater to ensure that specific behaviors of individual children are accurately captured by the instrument. The lack of training and communication can lead raters to attribute certain behaviors to incorrect behavioral domains of measurement (e.g., mistaking non-compliance for lack of attention), leading to unwanted error and decreased sensitivity to change.

Electronic ecological momentary assessment (eEMA) is a measurement method with characteristics that may improve the validity and reliability of reporting on behaviors commonly associated with IDDs. eEMAs encompass various techniques including diary recordings, experience sampling (i.e., multiple, randomly sampled time-points for observation or report throughout the day), and mobile- or web-based applications (Heron et al., 2017; Wilson et al., 2020; Thunnissen et al., 2022). eEMAs enable raters to directly report an individual’s behavior in near-real-time, across contexts, within flexible periods of time to establish an individual’s “typical behavior” based on both fluctuations and aggregates of all behavior recorded during the observation period (Heron et al., 2017).

The majority of EMA methods consist of self-report measures and are used as tools for typically developing (TD) individuals to explore a broad spectrum of human experiences including physical allergies (Schusteff et al., 2022), smoking cessation (Rowan et al., 2007) experience of psychiatric conditions (Raugh et al., 2019), and response to clinical treatment (Shiffman et al., 2008). Until recently, EMAs have excluded individuals with IDDs. These exclusions reflect the lack of self-report measures for individuals with IDDs in more traditional methodologies, and concerns related to reliability of self-reporting among these populations (Santoro et al., 2022). However a recent study by Wilson and colleagues demonstrated feasibility and reliability of a self-report experience sampling (i.e., external events, internal states, and emotions) eEMA measure piloted with 19 adults with mild to moderate intellectual disability (Wilson et al., 2020). In this study, participants received individualized training on how to use the eEMA mobile app, and practiced completing all items with study personnel and a caregiver before commencing their 7-day trial period. Participants received 7 randomly selected notification times to complete ratings each day. Participants completed on average 33.8% of their ratings. Split-half comparisons across all but one of the app’s 7 items indicated internal reliability; however, items were not validated against other established constructs or measures (Wilson et al., 2020).

In another set of studies, Ness and colleagues evaluated the Janssen Autism Knowledge Engine (JAKE) for use as an outcome measure in clinical trials with 29 youth diagnosed with autism (Ness et al., 2017). Parents rated a subset of customizable questions derived from the Autism Behavior Inventory (ABI) twice-weekly, allowing for an analysis of day-to-day fluctuations in autism-specific behaviors (Ness et al., 2019). However, compliance/use rates, feasibility, and reliability of the eEMA “daily tracker” were not reported (Ness et al., 2019).

The eEMA developed by our team improves areas of responding by directly addressing traditional response issues (Sudman et al., 1996) related to question interpretation (we provide training on behavioral domains and questions); information retrieval (we time-limit ratings to the current day); judgment formation (we carefully calibrate specific behavioral examples provided to the rater during training), response formatting (we include clear anchoring terms with each question), and response editing (we allow raters to return to previous items during their rating).

In the present pilot, proof-of-concept study, we sought to describe the content and function of *iBehavior*, a caregiver-report eEMA tool developed by our team, to assess and track behavior in children with IDDs, and to examine its preliminary functionality focusing on 10 families and a select set of behavior domains across a 14-day observation period. Though we employed a small sample in the present study, we provide an initial overview of *iBehavior’s* feasibility and early validity which will be expanded upon in a larger study that will include more comprehensive reliability and validity data.

## 2. Methods

All procedures performed in this study involving human participants were in accordance with the ethical standards of the institutional and/or national research committee and with the 1964 Helsinki Declaration and its later amendments or comparable ethical standards. The study was approved by the Institutional Review Board at University of California, Davis (No. 1865834).

### 2.1. The *iBehavior* mobile application

*iBehavior* is a smartphone-based (iPhone or Android) eEMA app designed for use by caregivers to assess problematic and social behaviors of people with neurodevelopmental disabilities, with a focus on its future application as an outcome measure for clinical trials. *iBehavior* content and function development was informed by a panel of stakeholders (parents, teachers, clinicians, researchers, and an FDA representative) invested in the assessment and treatment of children with IDDs, and by a Delphi study focused on Down syndrome, fragile X syndrome, and autism. After a review of currently available behavior outcome measures used in the field and review of an IDD caregiver survey that identified high-frequency behaviors, panel members anonymously submitted nominations of behavioral domains most critical to cover for each disability, based on their research, teaching, and clinical experience. Sixteen of the 19 panel members then completed an anonymous survey question, based on the nominated domains: “From this list of behavior problems, please rank ten from the most (1) to the least (10) impactful on daily functioning of individuals…” [with the particular disorder and age group]. The Delphi panel discussed the findings and reached consensus on the following domains for inclusion in *iBehavior*: (1) Irritable Mood and Aggression, (2) Avoidant, Fearful and Nervous Behavior, (3) Inattentive Behavior, (4) Stereotyped and Repetitive Behaviors and Interests, (5) Social Initiation and Responsiveness, and (6) Hyperactivity and Impulsivity (see Table 1). For the purposes of the present pilot study, the Hyperactivity and Impulsivity and Inattention items were omitted from data collection and analysis as the questions in these domains are now embedded within an *Executive Function-Related Behaviors* domain, which is currently being examined in a larger study.

**Table 1:** Means and standard deviations of *iBehavior* domains.

*iBehavior* domain	Mean	SD
Aggression and irritability frequency	0.42	0.54
Aggression and irritability intensity	0.43	0.27
Restricted and repetitive behaviors frequency	0.63	0.50
Restricted and repetitive behaviors intensity	0.58	0.42
Avoidant and fearful frequency	0.16	0.16
Avoidant and fearful intensity	0.17	0.17
Social initiation frequency	1.97	0.77
Social initiation intensity	2.07	0.88
Hyperactivity	*Domain not included in the present study*	
Inattention	*Domain not included in the present study*	

The *iBehavior* app was built using Nativescript, a framework for creating native mobile applications that targets multiple platforms (i.e., android and iOS). Data collected through the *iBehavior* app is encrypted and securely transmitted in real time to REDCap (Research Electronic Data Capture; Harris et al., 2009, 2019), which for this study is hosted and managed by the UC Davis Clinical and Translational Science Center (CTSC). REDCap database linkage is established via a study code which links their device to a REDcap database using REDCap’s application programming interfaces (APIs).

No personal identifying information (PHI) is recorded within the app or transmitted from the user’s device to REDCap. *iBehavior* content (domain items, language, scaling) can be individually added and/or modified in real time and pushed to the app for users. This feature enables the study team to field test content and respond to feedback in a timely manner. *iBehavior* also includes automatic, configurable notifications sent via SMS text messaging to remind users to begin their observations, and when to complete ratings. The connection between *iBehavior* and the REDcap database allows for real-time changes to app content on users’ devices by directly modifying the instruments in the REDcap database. The app is also remotely configurable, so that users can receive customized behavioral domain batteries that are specifically related to their needs, or the needs of researchers or clinicians. Individual behavior domains can also be turned “on” and “off” by study personnel through REDCap, and then be “pushed” to users in real time.

Currently, each *iBehavior* domain includes 6–8 items representing discrete types of behavior. For each behavior, the user is prompted to answer a yes/no question about whether the behavior occurred during the observation period. If “yes,” the app prompts the user to record the frequency (“rarely,” “sometimes,” “often,” or “very often”) and intensity (i.e., the degree of interference with daily functioning; “minimal,” “mild,” “moderate,” or “severe”) of the behavior. A key strength is that anchoring text accompanies, and is specific to each behavioral item, and provides specific information to guide a reliable rating (e.g., number of instances of the behavior; duration; degree of interference or distress). Many domain items were derived from existing instruments with strong item psychometric properties/factor loadings and clinical relevance for IDD populations (thus leveraging prior work).

In addition to specific behavior domains, *iBehavior* also includes situational questions, which are always presented before any other ratings can be made. The situational questions include general aspects of the observation period and the child’s health that day, the total time (in hours) the rater observed their child (0–24 h), the primary location of observation (I.e., home, school, work, traveling, vacation, in public, or other), whether the child was physically sick, and two questions regarding the quality of the child’s sleep the night prior.

### 2.2. Participants

Participants included 10 parents of children with IDD. Informed consent was obtained from all individual participants included in the study. All users identified as biological mothers, and nine were married. All parents graduated from high school, and seven earned a bachelor’s degree or higher. The children (2 females, 8 males) were between the ages of 8–17 years and were diagnosed with Down syndrome (DS; *n* = 3) and fragile X syndrome (FXS; *n* = 7). All children had intellectual disability, with nine previously documented by our study team using the Stanford-Binet 5th Edition (SB5), with full scale IQ deviation scores ranging from 31 to 68 (m = 57.1, sd = 14.1) and significant delays in adaptive behavior.

Unlike most other caregiver and teacher behavior rating scales, *iBehavior* requires rater training. This training includes three components: (1) technical set-up, functions and use of the *iBehavior* app, (2) explanation of eEMA and purpose of this method, and (3) a calibration interview with the rater focused on understanding the behavior domains, applying them to the child, how to judge intensity and frequency, and how the child’s specific behaviors should be recorded (e.g., confirmation of domains, clarification of level of intensity per example provided by the rater). During the training, the rater can also decide which days and times the app will notify them for observation.

### 2.3. Procedure

To begin, caregivers received one-on-one *iBehavior* training with trained staff via videoconference. Training lasted approximately 1 h and consisted of three components. First, parents were guided to install the app, and set up their child as a participant. Next, parents were trained on the structure of the app, how eEMAs are conducted, and how to approach frequency and intensity ratings of behaviors. Parent users also specified two times they preferred to receive text message reminders to begin and end daily observations (generally shortly after waking up and late in the evening after all contact with their child). Finally, parents participated in a calibration interview, in which behaviors across each domain were explored. Parents provided examples of their child’s behaviors they thought aligned with behaviors presented in the app, and were encouraged to ask questions and elaborate on their responses. If parents identified behaviors that did not align with those in the domain, the trainer redirected interpretation of their child’s behavior and discussed specific areas of misalignment and scoring.

After training, parents completed 14 days of *iBehavior* observational ratings in the following domains: (1) Irritable Mood and Aggression, (2) Avoidant, Fearful and Nervous Behavior, (3) Stereotyped and Repetitive Behaviors and Interests, and (4) Social Initiation and Responsiveness.

During participants’ observation period, a study team member inspected the database at pre-selected time points (days 1, 5, 7, 10, 14) and monitored data logging. If a data point was missing, the day was assumed to be skipped, and the participant was contacted via e-mail or phone to encourage them to resume observation. If ratings were skipped, study personnel asked participants to compete additional days to obtain 14 complete ratings from each participant. At the conclusion of the observation, participants were asked to complete a set of validation measures, and a user feedback questionnaire.

### 2.4. Validation measures

The *Aberrant Behavior Checklist-Community* (ABC-C) (Aman et al., 1985) is a global behavior checklist used to measure maladaptive behaviors among individuals with IDDs. The ABC-C consists of 58 items that target five behavioral dimensions (irritability, hyperactivity, lethargy/withdrawal, stereotypy, and inappropriate speech). Participants were instructed to complete the ABC-C based on behavior observed during the 14 days of *iBehavior* ratings.

The *Behavior Rating Inventory of Executive Function 2* (BRIEF-2) (Gioia et al., 2015) is a 63-item proxy report of a child’s executive function and includes inhibition, shifting, emotional control, working memory, and planning and organization. Participants were instructed to complete the BRIEF-2 based on behavior observed during the 14 days of *iBehavior* ratings.

*User Feedback.* All participants completed a 15-question user feedback survey that included questions about ease of use, technological problems, preference of *iBehavior* to other traditional questionnaires, as well as other aspects of usability, relevance, and satisfaction.

## 3. Results

**Figure 1:**
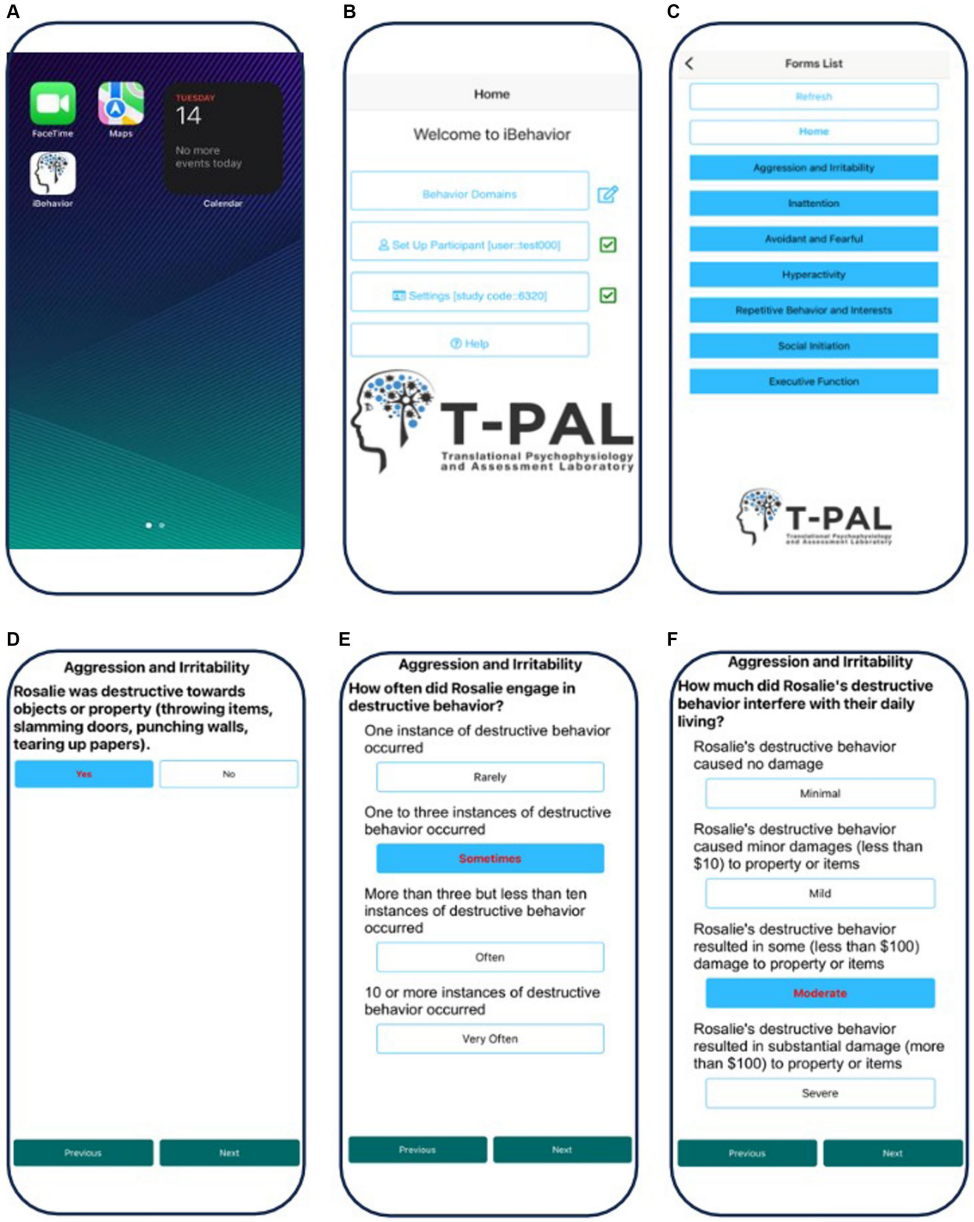
Screenshots of the *iBehavior* application on an iPhone. **(A)** Selection of the App on the home screen. **(B)** Home page of the app and selection of the behavior domains. **(C)** Selection of the specific behavior domain the user is rating (aggression and irritability). **(D)** Initial Yes or No answer selection indicating whether the behavior occurred during the observation period. **(E)** Rating the frequency of the behavior, with anchoring terms. **(F)** Rating the intensity of the behavior, with anchoring terms.

**Figure 2:**
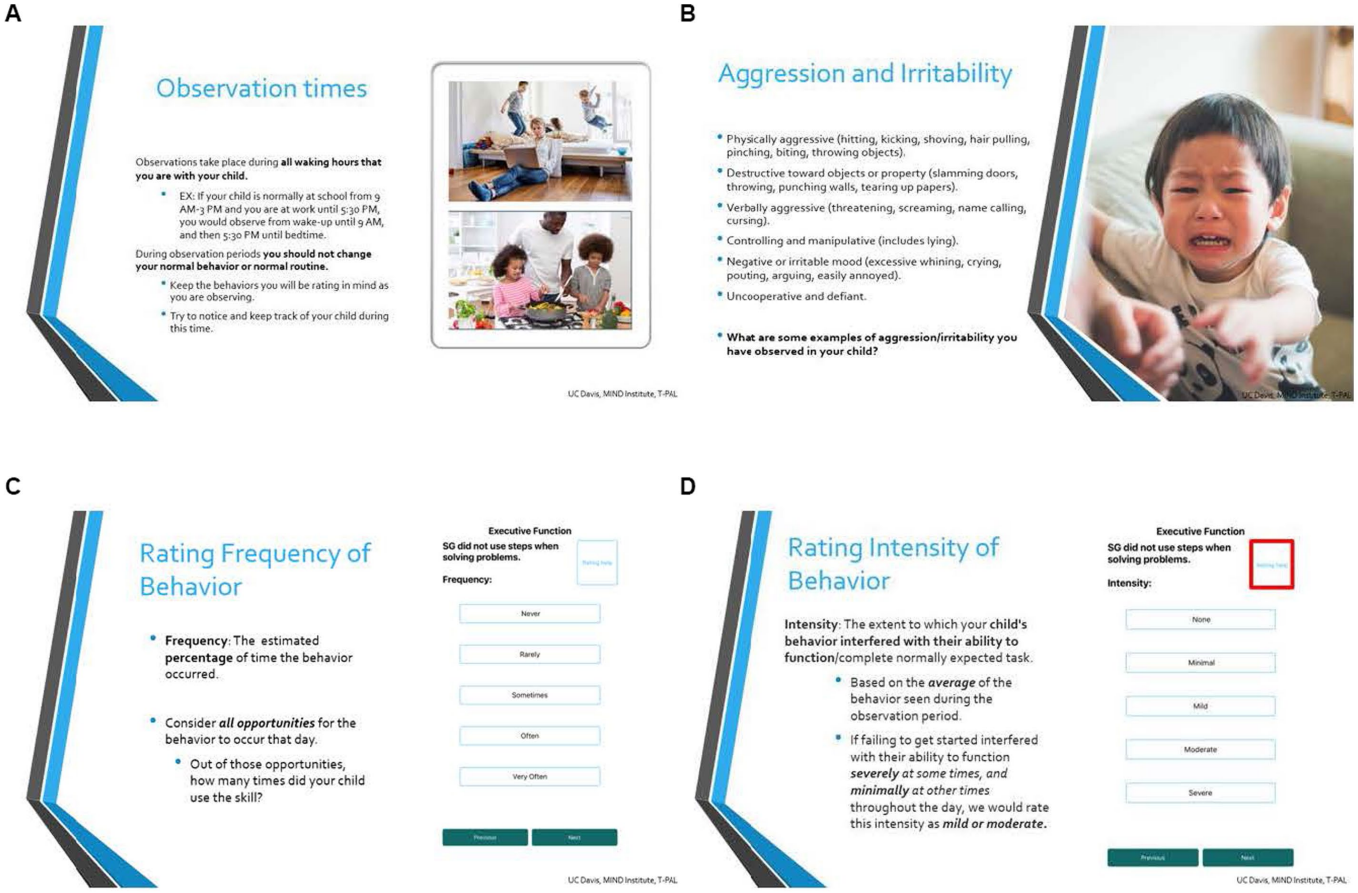
Select slides from *iBehavior* user training. **(A)** Introduces observation times and clarifies when the caregiver is responsible for recording behavioral data. **(B)** Discusses domain specific behaviors in relation to the participant’s child. **(C,D)** Calibrates the user to intensity and frequency ratings, respectively.

In order to examine feasibility of iBehaivor, participant observation logging was assessed. We also assessed preliminary content validity by examining relations between *iBehavior* domains and domains from the ABC-C and BRIEF-P (see Table 2). A rater feedback survey was conducted to examine ecological validity and feasibility (Figures 1, 2).

**Table 2:** Associations between *iBehavior* domains and validity measures.

	*iBehavior* frequency (F) and intensity (I) domains								
Variable	*df*	Aggression/Irritability F	Aggression/Irritability I	Avoidance, fearfulness, nervousness F	Avoidance, fearfulness, nervousness I	Repetitive behavior F	Repetitive behavior I	Social initiation F	Social initiation quality
BRIEF-2 inhibit	8	0.089	0.034	0.379	0.208	0.557	0.526	−0.367	−0.391
BRIEF-2 self-monitor	8	0.057	0.032	0.233	0.164	0.592	0.705*	−0.642*	−0.629
BRIEF-2 shift	8	0.144	0.050	0.573	0.474	0.817**	0.835**	−0.536	−0.399
BRIEF-2 emotional control	8	0.868**	0.807**	0.740*	0.691*	0.257	0.257	−0.434	−0.391
BRIEF-2 initiate	8	−0.322	−0.322	−0.305	−0.449	0.075	−0.069	−0.199	−0.187
BRIEF-2 working memory	8	0.037	−0.074	0.330	0.110	0.294	0.128	−0.422	−0.477
BRIEF-2 plan/organize	8	−0.549	−0.469	−0.440	−0.287	−0.557	−0.526	0.183	0.404
BRIEF-2 organization	8	0.083	−0.040	0.234	0.135	−0.049	−0.209	−0.529	−0.320
ABC-C irritability	8	0.735*	0.613	0.553	0.377	0.249	0.109	−0.237	−0.371
ABC-C social withdrawal	8	−0.079	−0.195	0.036	−0.055	0.377	0.365	−0.517	−0.578
ABC-C hyperactivity	8	0.347	0.334	0.418	0.479	0.285	0.479	−0.600	−0.491
ABC-C stereotypy	8	−0.341	−0.323	−0.128	−0.018	0.498	0.742*	−0.298	−0.164
ABC-C inappropriate speech	8	−0.411	−0.436	0.086	−0.049	0.483	0.373	0.275	0.324

*p < 0.05, **p < 0.01, ***p < 0.001.

### 3.1. Observation logging

Participants completed observations and recorded *iBehavior* data on an average of 13.3 of 14 days (range = 12–14). In total, five participants did not skip a single observation. Three participants skipped their last one or two observations, and only two participants skipped a rating in the middle of their observation window, but resumed ratings the following day after an email or phone call reminder. Across the 140 possible observations, 8 were skipped, leading to a 94% response rate over 10 participants’ observation periods. Participants also completed 100% of items for each of their logged observations.

### 3.2. *iBehavior* scores

To obtain domain summary scores, all items within their respective domain were summed over each observation day, generating a *total* domain score for each day. If participants indicated that the behavior in the item of interest was not present, a score of zero was assigned, thus, higher scores represented both greater frequency *and* intensity for the behavior, except for the social initiation and responsiveness domain, in which higher scores indicated *better* social initiation and responsiveness. Next, for each domain, total item scores for each day were summed, and then divided by the total number of observation days for each participant to account for differences in number of days observed across the sample. This procedure generated eight total scores, consisting of both frequency and intensity scores for each domain (means and standard deviations are reported in Table 1).

### 3.3. Convergent validity

Due to the small sample size, Spearman rank-order correlations were conducted to assess associations between frequency and intensity ratings for each of the four domains of *iBehavior*, and validation measures (i.e., ABC-C, BRIEF-2). For the ABC-C, the irritability sub-scale was significantly correlated with *iBehavior* irritable mood and aggression frequency [*r*(8) = 0.735, *p* = 0.015]. Additionally, the ABC-C stereotypy domain was significantly correlated with *iBehavior* stereotyped and repetitive behaviors and interests intensity [*r*(8) = 0.742, *p* = 0.014]. There were also associations with moderate correlation coefficients that did not reach statistical significance. For instance, on the ABC-C, the irritability sub-scale was related to *iBehavior* irritable mood and aggression intensity [*r*(8) = 0.613, *p* = 0.060] the lethargy and social withdrawal subscale was negatively associated with both social initiation frequency [*r*(8) = −0.517, *p* = 0.126] and intensity [*r*(8) = −0.578, *p* = 0.080].

For the BRIEF-2, emotional control was associated with *iBehavior* irritable mood and aggression frequency [*r*(8) = 0.868, *p* = 001] and intensity [*r*(8) = 0.807, *p* = 005], as well as *iBehavior* avoidance, fearfulness and nervousness frequency [*r*(8) = 0.740, *p* = 014] and intensity [*r*(8) = 0.691, *p* = 0.027]. In addition, BRIEF-2 shifting was associated with *iBehavior* stereotyped and repetitive behaviors frequency [*r*(8) = 0.817, *p* = 0.003] and intensity [*r*(8) = 0.835, *p* = 0.004].

### 3.4. User feedback

**Figure 3:**
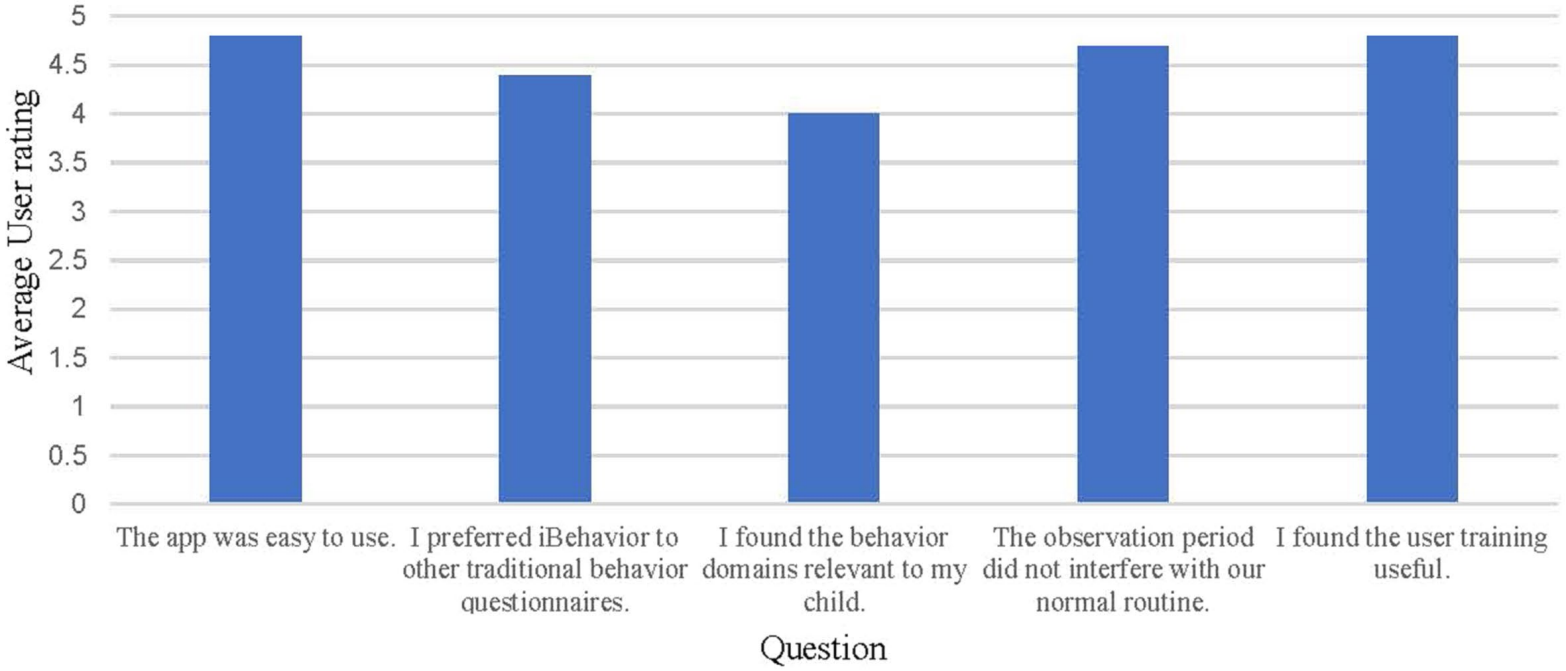
Average user responses to the *iBehavior* feedback questionnaire, completed at the end of user’s 14-day observation period. Users rated the questions on a Likert scale with the following response options: Strongly disagree (1), disagree (2), neither disagree or agree (3), agree (4), and strongly agree (5).

Caregivers were positive about their experience using *iBehavior*. On a five-point Likert scale ranging from (1) strongly disagree to (5) strongly agree, participants generally indicated high satisfaction with the app (see Figure 3). There were technical issues regarding text reminders being sent, and being sent at the correct times. These issues originated from errors in database set-up, and were resolved, however participants’ satisfaction with text reminders reflected these challenges (*m* = 2.5).

## 4. Discussion

The purpose of the present study was to describe the development and method, and examine the preliminary functionality and feasibility of *iBehavior*, a caregiver-report eEMA tool that is used to assess and track behavior in children with IDDs. Results of the present study provide preliminary evidence that *iBehavior* is a feasible behavioral outcome measurement tool in IDDs, and may offer important advantages over traditional retrospective rating scales, in particular, due to its ability to record behaviors shortly after they occur in context.

*iBehavior* demonstrated promising feasibility, given a 94% response rate over 10 participants’ observation periods across 14 days of observations. Participants also completed 100% of items for each of their logged observations. These results indicate that *iBehavior* is a feasible tool for capturing successive behavioral observations over multiple days. In addition, parents expressed overall high satisfaction using *iBehavior*, and specifically highlighted that the individual training component of using the app and making ratings was helpful. Although participants expressed neutral satisfaction with the text message reminder aspect of the app, this rating was likely driven by technical issues that were encountered during data collection, which were subsequently remedied.

Additional well-used and validated behavioral measures including the ABC-C, and BRIEF-2 indicated early evidence of convergent validity for *iBehavior* sub-domains. The aggression and irritable mood, stereotyped and repetitive behaviors, avoidance, fearfulness and nervousness, and social initiation domains aligned well with respective domains in the ABC-C (i.e., irritability, stereotypy, and lethargy/social withdrawal).

For the BRIEF-2 emotional control was related to both aggression and irritability and avoidance, fearfulness, and nervousness domains in *iBehavior*. This result is not entirely unexpected given the inherent externalizing behavioral aspects of both domains that *iBehavior* is designed to capture. These preliminary associations promote *iBehavior’s* behavioral sensitivity, and its ability to detect behavior frequency and social quality in the daily lives of children with IDD. Across these findings, a subsequent larger-scale study and sample will help us to further refine and develop items within behavioral domains.

We hypothesize that eEMA, compared to traditional rating scales, will reduce the contribution of expectancy bias to placebo responding (Erickson et al., 2017; Heneghan et al., 2017; Berry-Kravis et al., 2018). We expect that this will occur because, instead of rating behaviors in the research clinic (for example at the end of each treatment period), caregivers will instead rate behaviors more objectively due to the proximity of time and place that the behaviors occur. This hypothesis will be evaluated in an ongoing double blind, placebo-controlled crossover trial of liquid methylphenidate in 68 children and adolescents with IDD and comorbid ADHD (NCT05301361, Sensitivity of the NIH Toolbox to Stimulant Treatment in Intellectual Disabilities). If this and future trials confirm this hypothesis, several advances in clinical research may occur. First, reduced placebo responding and improved precision will increase statistical power to detect intervention benefits and reduce sample sizes needed for a desired effect size. Second, more frequent recording of key behaviors may help to reveal dynamic changes in behavior over time. Third, because user training includes discussion of each child’s unique behavior and how those behaviors are to be captured by the app, users may feel that their responses more accurately reflect their child, perhaps increasing accuracy and compliance.

This study was not without limitations. First, given the small number of participants, correlations between *iBehavior* domains and validation measures should be interpreted cautiously. Upcoming studies, including the aforementioned clinical trial and a larger feasibility study (*n* = 120) will be more adequately powered, particularly to assess reliability and validity. Second, all participants identified as biological mothers of the child they were observing, excluding fathers as reporters. Third, satisfaction and compliance of parents may be positively biased as those with interest in this technology or who feel negatively about traditional scales may be more likely to participate.

### 4.1. Conclusion

Results of the present pilot study indicate successful implementation and preliminary feasibility and validity of an eEMA tool for use as a behavioral outcome measure in IDDs. We found evidence for convergent validity among behavior domains and established caregiver measures on the ABC-C and BRIEF-2. Results from the user experience survey indicated satisfaction with the *iBehavior* app, and a preference to use it over traditional behavioral questionnaires. The creation of a secure smartphone-based eEMA measurement tool with targeted behavioral items specific to populations with IDDs is an innovative approach to detect treatment sensitivity, and with further validation it may serve as an important advancement in the field.

## Data availability statement

The raw data supporting the conclusions of this article will be made available by the authors, without undue reservation.

## Ethics statement

The studies involving humans were approved by the University of California, Davis Institutional Review Board. The studies were conducted in accordance with the local legislation and institutional requirements. Written informed consent for participation in this study was provided by the participants’ legal guardians/next of kin.

## Author contributions

AD contributed to writing the manuscript, data collection, and conducted statistical analyses. DG contributed to writing the manuscript and coordinating the study and data collection. HS contributed to the development of the app. MB developed iBehaivor. DH contributed to writing the manuscript and created *iBehavior* and obtained funding. All authors were involved in the creation and development of the content of *iBehavior*, as well as the tool itself.
