# Supplementary material for: iBehavior—a preliminary proof of concept study of a smartphone-based tool for the assessment of behavior change in neurodevelopmental disabilities
Source: Front Psychol. 2023 Oct 18;14:1217821. doi: 10.3389/fpsyg.2023.1217821 (PMC10619652; doi:10.3389/fpsyg.2023.1217821)
Supplement: Supplementary file 1 [file Data_Sheet_1.docx]

Supplementary Material

iBehavior - A Smartphone-Based Ecological Momentary Assessment Tool for the Assessment of Behavior Change in Neurodevelopmental Disorders

**Andrew Dakopolos, PhD^1+^ , Dana Glassman, BA^1+^ , Haleigh Scott, PhD^1^ , Michael Bass, MS^2^ &**

**David Hessl, PhD^1^ ***

*** Correspondence:** David Hessl, PhD Director, Translational Psychophysiology and Assessment Laboratory, MIND Institute, UC Davis Medical Center: [drhessl@ucdavis.edu](mailto:drhessl@ucdavis.edu)

# Supplementary Data

iBehavior Behavioral Stems:

Aggression and Irritability:

- Was physically aggressive towards other people (hitting, kicking, shoving, scratching, hair pulling, pinching, biting, throwing objects).
- Was destructive towards objects or property (throwing items, slamming doors, punching walls, tearing up papers).
- Was verbally aggressive towards others (threats of harm, screaming, name calling, cursing).
- Was controlling and manipulative (trying to force will on others, lying).
- Was in a negative or irritable mood (excessive whining, crying, pouting, arguing, easily annoyed).
- Was uncooperative and defiant (refusing to comply, rebellious, disobedient, throwing self to floor).

Inattention:

- Was unable to follow or forgot instructions/directions (not due to being oppositional or a failure to understand).
- Had difficulty paying attention to verbal, visual, or written information.
- Avoided, showed reluctance, or complained about tasks or activities requiring sustained attention or consistent effort (chores, homework, etc.).
- Was distracted from school tasks, chores, play or activities.
- Was messy, disorganized, or forgot needed items in work, school, or other activities.
- Had difficulties with tasks that required attention to detail and/or made careless mistakes at work, school, chores, or other activities.
- Had difficulty concentrating on or finishing work, school, chores, or other tasks.

Avoidance, Fearfulness, and Nervousness:

- Showed fear of or avoided a specific place, object or animal/insect.
- Showed fear of, avoided, or withdrew from people, or was visibly nervous/distressed in social groups or interaction (NOT only a lack of interest in people).
- Made statements or vocalizations reflecting worry, apprehension, distress or fear.
- Showed fear/nervousness about change, loss of routine, or disruption of desired activity.
- Expressed distress or fear (e.g., crying, clinging, refusal) about being separated from a caregiver, and/or showed clinginess/reluctance to be alone or sleep alone.
- Showed any physical signs of anxiety/fear/nervousness: visibly trembling, shaking, sweaty, difficulty breathing, physically tense, restless, pacing, easily startled, frequent urination, stomach upset that was not captured in previous questions.

Hyperactivity:

- Was overly active (physically moving too much, often on the go, moving too quickly from one activity to another).
- Was impulsive and/or acted without thinking.
- Was restless, fidgety, or squirmy.
- Talked too much (blurted out, interrupted or bothered others with talk).
- Had difficulty waiting.
- Had difficulty remaining still or seated when appropriate (meals, lessons, etc.).

Repetitive Behaviors and Interests:

- Engaged in repetitive body movements (e.g., rocking, pacing, hand-flapping).
- Used repetitive, scripted, or echoed language (e.g., from a movie or song).
- Used or played with objects in an odd or repetitive manner (spinning or lining up objects).
- Had difficulties with changes to routines or planned events.
- Showed an overly intense or odd interest on particular topics or activities.
- Was physically harmful to themselves.

Social Initiation

- Started a social interaction such as conversation or play with another person.
- Participated in back-and-forth social interaction such as a conversation or interactive play with another person.
- Responded to the emotions of others or expressed their own emotions to others.
- Shared his/her own interests or experiences with another person (showing, pointing, sharing an event or object, etc.).
- Had social interactions with peers.
- Thinking about all of XXX’s social contact during the observation period, how was their use of physical means of nonverbal communication (Eye contact, facial expressions, gestures)?
- Thinking about all of XXX’s social contact during the observation period, how was their use of vocal nonverbal communication (tone and volume)?

**
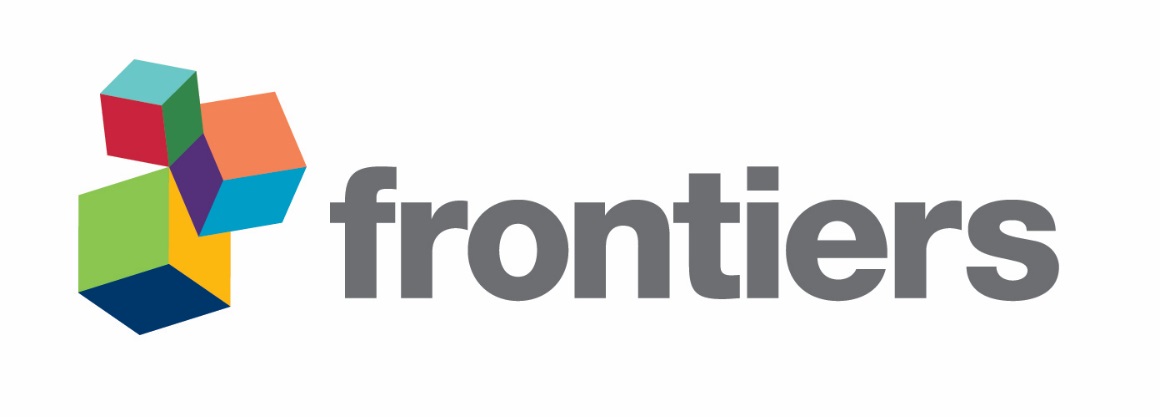
**
